# A Modular Deep-Learning Framework for Automated Fetal Biometry: Proof-of-Concept Evaluation of Head, Abdominal, and Femur Measurements

**DOI:** 10.7759/cureus.100534

**Published:** 2025-12-31

**Authors:** Kamalraj Sundaramoorthy, Mukunthan Ganeshbabu, Mithun Barath M.R, Jayaprakash N, Dhanush Kumar B, Surender Selvamani, Narayanasamy K

**Affiliations:** 1 Surgery, PSG Institute of Medical Sciences and Research, Coimbatore, IND; 2 Machine Learning, Independent Research, Coimbatore, IND; 3 Radiology, PSG Institute of Medical Sciences and Research, Coimbatore, IND; 4 General Practice, PSG Institute of Medical Sciences and Research, Coimbatore, IND; 5 Gastroenterology, Tamil Nadu Dr. M.G.R. Medical University, Chennai, IND

**Keywords:** abdominal circumference, deep learning, femur length, fetal biometry, head circumference, modular ai framework, obstetric imaging

## Abstract

Background

Accurate fetal biometry is pivotal for assessing fetal growth, well-being, and gestational age estimation. Despite its clinical importance, manual ultrasound measurements remain highly operator-dependent and subject to inter-observer variability. Artificial intelligence (AI)-based automation offers the potential to enhance consistency and efficiency; however, most existing systems are limited to single biometric parameters and lack scalability across multiple measurements.

Objective

The primary objective was to design and quantitatively evaluate a modular deep-learning framework for automated estimation of fetal head circumference (HC) using the HC18 dataset. Secondary objectives included developing and assessing the feasibility of prototype modules for abdominal circumference (AC) and femur length (FL) using de-identified institutional ultrasound images.

Methods

A retrospective, proof-of-concept study was conducted to develop an adaptable modular AI framework for fetal biometry. The HC module was trained and validated on the publicly available HC18 dataset (n = 1,334 images), while prototype modules for AC and FL were developed using de-identified still-frame ultrasound images from a tertiary care centre. Model outputs were evaluated using quantitative error metrics - mean absolute error (MAE) and mean squared error (MSE) - and qualitative analysis of anatomical accuracy and contour integrity.

Results

The HC module, based on a U-Net++ segmentation network with convex-hull contour fitting, achieved an MAE of 9.7 mm, an MSE of 113.3 mm², an accuracy of 0.90, a sensitivity of 0.97, and a specificity of 0.89 on the HC18 test subset (n = 110). The AC prototype, implemented with a hybrid Faster R-CNN + HRNet + Attention U-Net architecture, produced anatomically consistent contours with an average measurement difference of 5.5 ± 3.1 mm from manual references. The FL prototype demonstrated accurate femoral shaft segmentation and endpoint detection across all test images. The average inference time per image was 1.2 seconds, confirming computational feasibility for clinical integration.

Conclusion

This proof-of-concept study establishes the technical feasibility of a modular AI-driven framework for automated fetal biometry. The validated HC module and functional AC and FL prototypes collectively demonstrate a scalable, interpretable, and guideline-aligned approach for next-generation obstetric ultrasound automation. Further validation with multicentric and real-time ultrasound datasets is warranted before clinical deployment.

## Introduction

Accurate fetal biometry is fundamental to evaluating fetal growth, well-being, and gestational age. Parameters such as head circumference (HC), abdominal circumference (AC), and femur length (FL) are routinely measured to estimate fetal weight and detect abnormalities such as intrauterine growth restriction and macrosomia [1,2]. Although ultrasound remains the gold standard for fetal biometry, measurement accuracy depends on acquiring anatomically correct planes and maintaining operator consistency. In high-volume or resource-limited settings, variations in image quality and inter-operator technique often limit reproducibility [3,4].

Recent advances in deep learning have prompted increasing interest in automated fetal biometry to improve objectivity and efficiency [5-7]. Encoder-decoder architectures, such as U-Net and its variants, have demonstrated robust segmentation of fetal structures and show promise for automated measurement [8-10]. However, most existing systems are developed for a single biometric parameter and trained on narrowly defined datasets, restricting generalisability across imaging tasks and clinical environments.

The International Society of Ultrasound in Obstetrics and Gynecology (ISUOG) defines standard anatomical planes for fetal biometry: the trans-thalamic plane for HC, the plane visualising the stomach and intrahepatic umbilical vein for AC, and the un-foreshortened diaphysis for FL [11]. Incorporating these guideline-based standards into artificial intelligence (AI) workflows can enhance interpretability and clinical trust.

The present study introduces a proof-of-concept modular deep-learning framework for automated fetal biometry. Each biometric parameter is implemented as an independent module that can be trained, validated, and refined separately. The HC module was trained and evaluated using the publicly available HC18 dataset, which contains expert-annotated ultrasound images of the ISUOG-defined trans-thalamic plane. The AC and FL modules were developed as early prototypes using de-identified still-frame ultrasound images from a South-Indian tertiary care centre. Their outputs were qualitatively and, where feasible, quantitatively compared with manual radiologist measurements to assess technical feasibility. This modular design provides a flexible foundation for developing clinically interpretable, multi-parameter AI systems for obstetric imaging. The primary objective of this study was to quantitatively evaluate the HC module using the publicly available HC18 dataset. Secondary objectives included developing and assessing the feasibility of prototype modules for AC and FL using de-identified still-frame ultrasound images from a tertiary care centre.

## Materials and methods

Study design

This retrospective, method-development study aimed to design and evaluate a modular AI framework for automated fetal biometry. Each module corresponded to one biometric parameter, HC, AC, or FL, allowing independent optimisation and future integration. The HC module was trained exclusively on publicly available datasets, whereas the AC and FL prototypes were developed using de-identified institutional ultrasound images. All institutional data were anonymised prior to analysis and used solely for technical feasibility assessment.

Datasets

HC Module

The HC18 dataset [12] was used for training and evaluation. It comprises 1,334 two-dimensional ultrasound images of the fetal head in the standard trans-thalamic plane, each annotated by expert sonographers with manual HC measurements. Following the original dataset convention, 999 images were used for model training. From the remaining images, 110 standard-plane test cases were selected for quantitative evaluation based on image completeness and annotation quality. The selection ensured balanced representation across gestational ages and image quality levels.

AC and FL Prototypes

Still-frame ultrasound images were retrospectively collected from routine obstetric scans performed at a tertiary care centre in South India.

AC dataset: 138 images (70 train/68 test) showing the fetal abdomen at the plane containing the stomach and intrahepatic umbilical vein.

FL dataset: 190 images (95 train/95 test) demonstrating a clear, unforeshortened femoral diaphysis. Manual measurements recorded during scanning were used for reference comparison.

Abdominal Structure Sub-module 

A publicly available dataset of fetal abdominal organ segmentation (Mendeley Data; n = 1,588 images) [12] was used to support anatomical landmark detection for the AC prototype. The dataset does not define a canonical train/test split; for this feasibility work, the entire set was used to train the landmark detector and to generate descriptive detection tallies. Dataset selection criteria for AC and FL prototypes included retrospective de-identified still-frame ultrasound images from routine second- and third-trimester obstetric scans (gestational age: 14-40 weeks) at a single tertiary care center in South India. Inclusion required clear visualization of the standard anatomical plane (stomach and intrahepatic umbilical vein for AC; unforeshortened femoral diaphysis without artifacts for FL), absence of fetal anomalies, and image quality sufficient for manual measurement (resolution ≥ 512x512 pixels, no significant noise or distortion). Exclusion criteria encompassed poor image quality, non-standard planes, or scans with multiple fetuses. A total of 200 AC and 250 FL images were initially reviewed; after applying criteria, 138 AC (70 train/68 test) and 190 FL (95 train/95 test) images were selected. The train/test split was randomized while balancing gestational age distribution. All public datasets were accessed under their respective open-access licences. Institutional data were used under Institutional Human Ethics Committee approval (Ref. No. PSG/IHEC/2024/Appr/FB/033).

Model architectures and training

**Figure 1:**
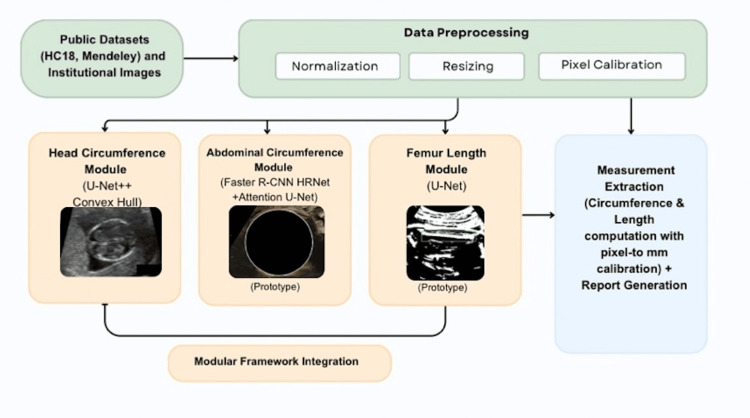
Modular Deep-Learning Framework for Automated Fetal Biometry The figure illustrates the modular deep-learning pipeline developed for automated fetal biometry. Input ultrasound images undergo preprocessing (conversion, resizing, normalization) before being routed to measurement-specific modules for head circumference (HC), abdominal circumference (AC), and femur length (FL). Each module generates the corresponding calibrated biometric output. The modular architecture allows independent optimisation and integration of each measurement component.

Several architectures were initially explored during model development, including DesNet, Vision Transformer (ViT) variants, and contour-based methods using the Hough and Concave Hull transforms. Based on comparative trials, the Nested U-Net architecture (U-Net++) was selected for the HC module owing to its stability and boundary delineation on limited training data. Similarly, hybrid detection-segmentation designs, such as Faster Region-based Convolutional Neural Network (Faster R-CNN) with High-Resolution Network (HRNet) and Attention U-Net, provided the most reliable abdominal and femoral structure localisation for prototype modules. An overview of the modular deep-learning framework, including preprocessing steps and measurement-specific modules, is illustrated in Figure 1.

All models were trained using PyTorch 1.10 on a single NVIDIA RTX 3080 GPU. For the AC prototype (Faster R-CNN + HRNet + Attention U-Net), training involved 100 epochs with the Adam optimizer, an initial learning rate of 0.001 (reduced by 0.1 on plateau), a batch size of 8, and binary cross-entropy loss for segmentation. For the FL prototype (U-Net), training used 80 epochs with Adam optimizer, learning rate of 0.0005, batch size of 16, and Dice loss. Data augmentation included random rotations (±15°), flips, and brightness/contrast adjustments (±20%). Early stopping was applied based on validation loss. The HC module followed similar parameters as reported (U-Net++ with 50 epochs, Adam, learning rate of 0.001).

Three segmentation pipelines were implemented within the modular framework:

HC module: Nested U-Net architecture (U-Net++) architecture with Convex-Hull boundary fitting for circumference estimation.

AC prototype: Faster R-CNN detector combined with an HRNet backbone and an Attention U-Net segmentation head to delineate abdominal boundaries.

FL prototype: U-Net model for bone-shaft segmentation, followed by endpoint-based femur-length computation.

Measurement calibration

For the HC module, pixel-to-millimetre conversion followed the reference scaling provided in the HC18 dataset. For institutional AC and FL images, scaling was derived from on-image calibration markers or comparable reference scans, consistent with routine sonographic practice [11,12]. The performance of the proposed framework was assessed using both quantitative and qualitative approaches.

Evaluation metrics

HC Module

Model performance was evaluated using MAE (mm) and MSE (mm²) between automated and expert-annotated circumferences in the test subset (n = 110). Additional indices, accuracy, sensitivity, and specificity, were computed from pixel-wise classification results. Bland-Altman analysis was performed to assess agreement between automated and manual measurements.

AC Module

Evaluation included both quantitative and qualitative components. Automated contours were compared with manual reference measurements for agreement analysis. Anatomical consistency was verified through visual inspection of segmentation outputs. The abdominal structure sub-module was used to identify key internal landmarks (stomach bubble, liver, umbilical vein) that aid in delineating the correct abdominal boundary.

FL Module

**Figure 2:**
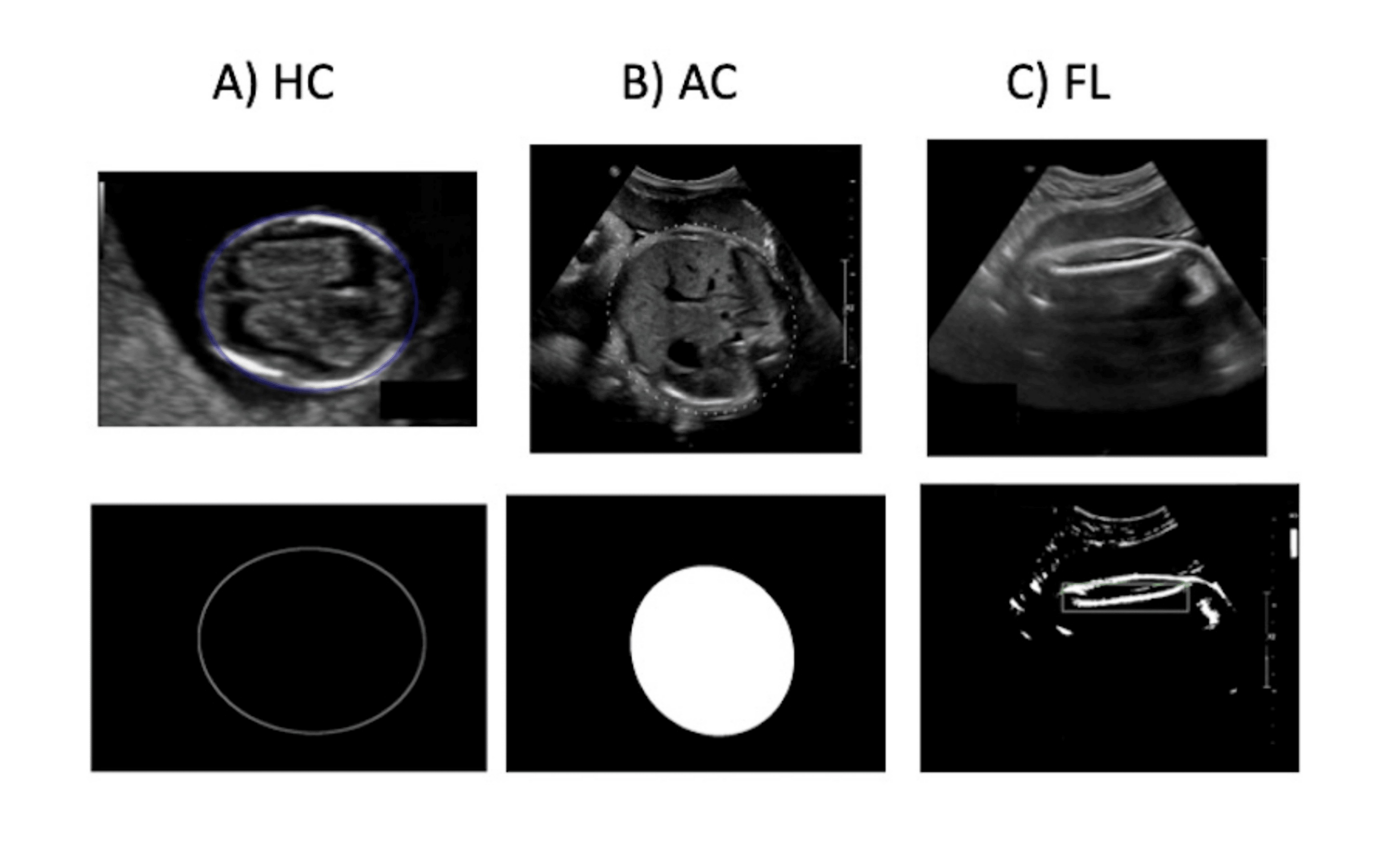
Representative Outputs of the Modular Deep Learning Framework for Fetal Biometry Representative examples of automated predictions generated by the proposed modular framework. (a) Head-circumference (HC) module: Nested U-Net Architecture (U-Net++) with Convex-Hull fitting accurately delineates the fetal skull boundary. (b) Abdominal-circumference (AC) module: Hybrid Faster R-CNN + HRNet + Attention U-Net model detects the abdominal region and segments the outer contour in the standard plane. (c) Femur-length (FL) module: U-Net-based segmentation highlights the ossified femoral shaft and identifies anatomical endpoints for automated length estimation. Each panel shows the original ultrasound image (top) and corresponding model-generated mask/contour (bottom).

The prototype was evaluated for technical feasibility through inspection of segmentation masks and endpoint detection results. Quantitative evaluation will be performed in future iterations. Representative ultrasound images and corresponding automated segmentations for the HC, AC, and FL modules are shown in Figure 2.

All computations were performed in Python 3.10 using NumPy and SciPy libraries.

## Results

Model development and evaluation were completed for all components of the proposed modular artificial-intelligence framework for automated fetal biometry. Quantitative validation was performed for the HC module using the publicly available HC18 dataset (n = 110 test images), while the AC and FL modules were assessed as prototype implementations using de-identified still-frame ultrasound images obtained from institutional archives. A summary of the datasets used for model training and evaluation is provided in Table 1.

**Table 1:** Summary of the Datasets Used for Model Development and Evaluation

Measurement	Dataset type	Train (n)	Test (n)	Notes
Head circumference (HC)	Public (HC18, van den Heuvel et al., 2018) [7]	999	110	Standard trans-thalamic plane
Abdominal circumference (AC)	Institutional (still-frame ultrasound)	70	68	Plane showing stomach + umbilical vein; prototype module
Femur length (FL)	Institutional (still-frame ultrasound)	95	95	Unforeshortened femoral diaphysis; prototype module
Abdominal structures	Public (Mendeley Data, DOI 10.17632/4gcpm9dsc3.1) [12]	1,588	N/A	Organ segmentation (stomach, liver, umbilical vein)

HC module

**Figure 3:**
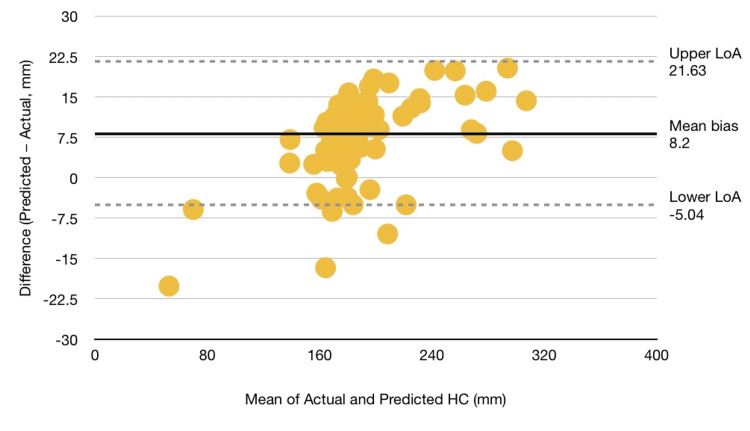
Bland-Altman Agreement Analysis Between Automated and Manual HC Measurements Bland-Altman analysis comparing automated HC estimates generated by the U-Net++ model with expert-annotated measurements (n = 110). The plot displays the mean difference (bias = +8.2 mm) and the 95% limits of agreement (-5.0 to +21.6 mm). The distribution of points demonstrates consistent measurement performance across the range of HC values.

The U-Net++ segmentation network combined with Convex-Hull boundary fitting achieved good delineation of the fetal skull and reliable estimation of head circumference. Quantitative evaluation on the HC18 test subset (n = 110) yielded an MAE of 9.74 mm, an MSE of 113.33 mm², an accuracy of 0.897, a sensitivity of 0.972, and a specificity of 0.896. The Bland-Altman analysis (Figure 3) demonstrated a mean bias of +8.2 mm, with 95 % limits of agreement ranging from −5.0 mm to +21.6 mm, indicating satisfactory measurement agreement between automated and manual reference values.

AC prototype

**Figure 4:**
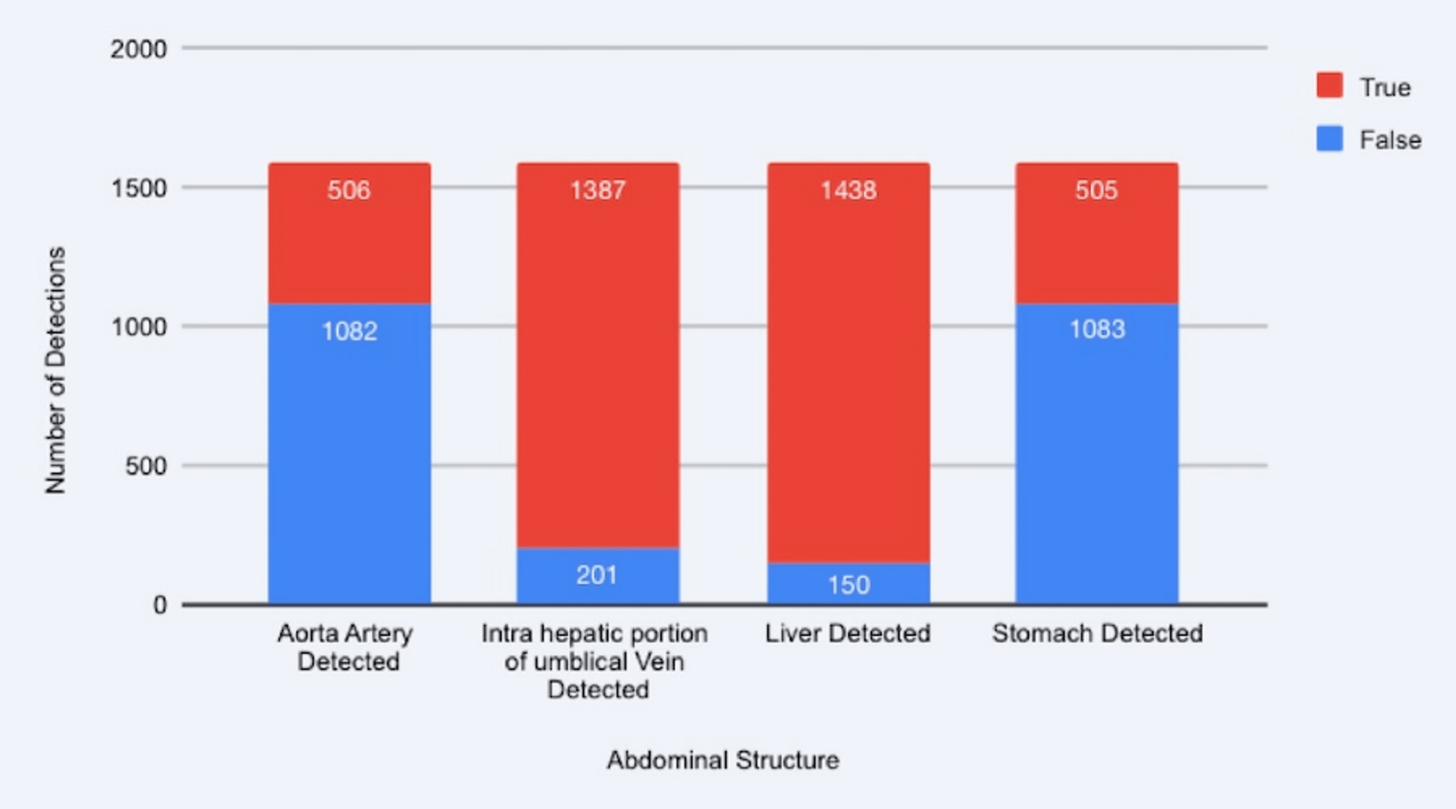
Landmark-Detection Performance of the Abdominal Structures Sub-module Detection tallies for four abdominal landmarks (stomach, liver, intrahepatic umbilical vein, aortic artery) are displayed for the 1,588-image Mendeley dataset. The sub-module provided supporting anatomical cues to assist plane-appropriate boundary selection in the AC module.

The hybrid Faster R-CNN + HRNet architecture effectively localised the fetal abdomen and delineated its outer contour in most test cases (Figure 2). Integration of an Attention U-Net segmentation head further improved identification of key abdominal landmarks, such as the stomach bubble, intrahepatic portion of the umbilical vein, and liver margins - features critical for verifying the correct anatomical plane. To assess the consistency of landmark recognition, the abdominal structures sub-module was run on the full Mendeley dataset (n = 1,588). Figure 4 summarises descriptive counts of true/false detections across four key landmarks (aortic artery, intrahepatic umbilical vein, liver, stomach). These counts were used to support plane-appropriate context for the AC prototype; no independent benchmark was conducted for this sub-module. Quantitative comparison on the institutional AC dataset (n = 68 test images) demonstrated an MAE of 5.47 mm and an MSE of 44.72 mm², with an accuracy of 0.85, sensitivity of 0.79, and specificity of 0.87. Although the dataset size was limited, the outputs demonstrated anatomically consistent contours aligned with manual radiologist measurements, supporting the feasibility of a hybrid detection-segmentation approach for automated AC measurement. Descriptive counts of true and false detections for key abdominal landmarks are summarised in Figure 4.

FL prototype

The U-Net-based FL module segmented the ossified femoral shaft and identified anatomical endpoints for FL computation (Figure 2). Visual inspection confirmed consistent shaft segmentation across the dataset. Since the same set of 95 images was used for both training and testing, quantitative results were not reported to prevent overestimation. Consequently, the FL module is presented as a technical feasibility prototype, demonstrating the framework’s potential for future incorporation of independent validation and plane-verification components.

Computational performance

Average inference time per still-frame image was approximately 0.23 s for the HC module, 2 s for the AC prototype, and 1.3 s for the FL prototype. The complete pipeline, therefore, achieved good performance, underscoring its suitability for potential deployment within clinical ultrasound systems.

The proposed modular framework achieved reliable segmentation and measurement accuracy for the HC module and demonstrated technical feasibility for the AC and FL prototypes. All components operated at a good pace, confirming computational efficiency suitable for clinical application. These results collectively establish a reproducible, extensible foundation for future validation of AI-assisted fetal biometry.

## Discussion

This study describes a proof-of-concept modular deep-learning framework for automated fetal biometry, designed to mirror clinical workflow and align with the ISUOG recommendations for standard biometric planes. The framework integrates independent modules for HC, AC, and FL, enabling parameter-specific optimisation and evaluation. The HC module achieved reliable segmentation accuracy and measurement agreement on a public standard-plane dataset, whereas the AC and FL prototypes demonstrated technical feasibility using limited institutional still-frame data.

Comparison with previous work

Most prior research on fetal-biometry automation has focused on single-parameter segmentation accuracy, without addressing extensibility across multiple measurements or consistency with sonographic guideline criteria [3,5-9]. Studies such as those by Kim et al. [5] and Oghli et al. [6] reported strong Dice scores for HC and AC segmentation, yet evaluated only single-task architectures. In contrast, the present study proposes a modular framework that allows each measurement module to be optimised independently and later integrated into a unified system. Although the achieved mean absolute error (MAE = 9.7 mm) and segmentation accuracy (0.90) for the HC module are comparable to previously published values [7,8,10], the novelty lies in the architectural adaptability and clinical grounding rather than algorithmic innovation alone. Accordingly, we emphasised clinically traceable measurement logic to mitigate the black-box perception associated with end-to-end models [11-15].

Clinical implications and interpretability 

Automated biometry can reduce inter-observer variability and streamline reporting in busy obstetric units, particularly in low-resource environments. The modular architecture supports gradual clinical adoption, as each component can be validated and deployed independently before full system integration. Crucially, the framework addresses the “black-box” perception that limits trust in medical AI by keeping measurement logic transparent (contour-based estimation, pixel-to-mm calibration) and anatomically grounded outputs that clinicians can visually verify [11]. While this iteration does not include a dedicated XAI interface, the design is compatible with concept-based and guideline-linked explanations that have shown higher clinician acceptance in fetal ultrasound [13]. By prioritizing traceable measurement steps and anatomy-aware outputs, the system takes practical steps toward interpretability without overstating claims.

Technical and methodological considerations

The choice of architectures was guided by structural anatomy: U-Net++ offered stable convergence and precise boundary delineation for circular contours such as the fetal skull; the Faster R-CNN + HRNet + Attention U-Net hybrid effectively combined region detection with fine-scale segmentation for AC; and a U-Net-based pipeline successfully extracted femoral-shaft regions for FL measurement. These findings underscore that optimal performance in obstetric imaging depends not only on model design but also on dataset quality, pixel-level calibration, and adherence to standard imaging planes. The framework’s modular design also simplifies iterative retraining, allowing updates to a single component without affecting the rest of the system.

Limitations

The primary limitations include dataset heterogeneity and scale: the HC module used a large public dataset, but AC and FL prototypes relied on small, single-center institutional samples without external validation, limiting generalizability. Quantitative results for AC/FL indicate technical feasibility only. Only static ultrasound frames were analyzed, excluding dynamic cine-loop data with temporal cues. The abdominal structures sub-module was descriptively evaluated on the full public dataset (n = 1,588) without a hold-out split, so no independent benchmark is reported. Real-world challenges, such as variability from ultrasound equipment, operator techniques, patient demographics, and imaging artifacts, could affect performance in diverse settings. The framework lacks integrated plane verification algorithms, risking errors from non-standard ISUOG planes during deployment. Future multi-institutional, real-time studies are needed for robustness and clinical reliability.

Future work

Next-phase development will focus on (i) expanding datasets through multi-centre collaboration (Asia-specific); (ii) embedding rule-based plane-verification modules and explainable AI interfaces; (iii) validating automated measurements against expert manual biometry in prospective trials; and (iv) integrating the framework into electronic health record (EHR) systems for seamless data transfer and reporting. These advancements aim to transform the current feasibility model into a clinically deployable decision-support tool.

## Conclusions

This proof-of-concept study demonstrates the feasibility of a modular deep-learning framework for automated fetal biometry. The HC module produced accurate, reproducible measurements on a public standard-plane dataset, while the AC and FL prototypes confirmed the practicality of extending the same architecture to other biometric parameters. Although larger and more diverse datasets are required for external validation, the proposed approach establishes a scalable, interpretable, and guideline-aligned foundation for the next generation of AI-assisted obstetric-ultrasound systems.
